# The Brain Power of Man

**Published:** 1874-06

**Authors:** 


### Our Exchanges.

---

THE BRAIN POWER OF MAN.—A very large and intelligent audience of ladies and gentlemen assembled in the Congregational Church, corner of Tenth and G streets, Washington, to hear the distinguished physician, Dr. Brown Sequard, of Boston, discuss the question, "Have we two brains, or only one? and if two, why not educate both?"

The lecturer was appropriately introduced by Prof. Henry, who stated that the lecture was one of a series generously provided for by Dr. J. M. Toner for the discovery of new facts in medicine.

Dr. Sequard commenced by saying that his views, he hoped, being somewhat novel, would command attention. The facts he would dwell upon were new, probably would not be generally accepted, and perhaps would not be easily understood by those not familiar with medicine.

Have we two brains? and, if so, why not educate both? The views of science upon this subject were different from his. The left side of the body was the side affording volition to the brain, and, *vice versa*, the right side of the brain afforded volition to the body. Eminent authorities had declared that either side of the brain was competent for this purpose. But we use only one side, and, therefore, leave out of account one-half of brain matter. We owe due education to both sides of the brain, or, rather, to the two brains. * * *

All this shows, not that the two sides of the brain differed originally, but that there were different developments of each. The left side of the brain was much larger than the right side. If a person went frequently to the same hatter, he would find that his hat had from time to time to be enlarged. There was no question that the brain grew. By studying a particular subject the person became more proficient, and the brain was more fully developed.

There was no doubt that the left side of the brain predominated in our system. Our being right-handed showed it. There was no population in the world that was not right-handed. The right hand of the body was mostly used. Left-handed individuals used the right side of the brain, showing the connection between these things.

There was primitively a difference between the two brains. In children convulsions were sooner developed in the left than in the right side of the brain. This was attributable to excess of blood in the left side. Parrots roosted on the right legs, and their talking power came from the left side of the head.

There were four vital points to be considered. The first was that asphyxia was connected with the left side of the brain in persons that were right-handed, and with the right side in those that were left-handed. The second point was that children who were first learned to talk, if disease came in the left side of the brain, learned to talk just as well with the right side of the brain. Though losing half of the brain, they got along just as well.

This proved that the right side could be educated, with the left hand for execution. The third point was, that four out of every hundred left-handed persons learned to write with the left hand; therefore the left side of the brain, even with persons left-handed, could be educated better than the right side. The fourth point was, that the leg was rarely so much affected by paralysis as the arm. He, however, would pass over this argument, as it could only be understood by medical men.

If the lecturer had established that we had two brains, then they should be developed. If we could develop the legs and the arms of both sides, we could develop both sides of the brain. If we gave as much attention to the left side of the body as we do to the right side, we would fully develop our two brains. The important point, therefore, would be to make children use both sides of the body—alternately using the right and left arm, and the right and left leg equally. There would be no difficulty in thus training children to full development.

Even adults who had lost speech by disease of the left side of the brain, could regain the power by cultivating the right side. In gesture, persons who had lost the use of the right arm could be trained to use the left. If children were thus trained we would have a sturdier and healthier race, both mentally and physically.

